# Meta-Analysis: Narrow Band Imaging for Diagnosis of Gastric Intestinal Metaplasia

**DOI:** 10.1371/journal.pone.0094869

**Published:** 2014-04-17

**Authors:** Jia Song, Jixiang Zhang, Jun Wang, Xufeng Guo, Jing Wang, Ya Liu, Weiguo Dong

**Affiliations:** Department of Gastroenterology, Renmin Hospital of Wuhan University, Wuhan, Hubei Province, P.R.C.; University Hospital Llandough, United Kingdom

## Abstract

**Background:**

Distinguishing early gastric cancer is challenging with current imaging techniques. Narrow band imaging (NBI) is effective for characterizing gastric lesions.

**Objectives:**

The aim of this meta-analysis was to estimate the diagnostic accuracy of NBI in the gastric intestinal metaplasia (GIM).

**Methods:**

We performed data analysis using Meta-DiSc (version 1.4) and STATA (version 11.0) software. To assess study quality and potential for bias, we used the Quality Assessment of Diagnostic Accuracy Studies-2 (QUADAS-2) tool.

**Results:**

Six studies involving 347 patients were included. On a per-patient basis, the sensitivity of NBI for diagnosis of GIM was 0.65 (95% CI  =  0.56–0.74), and the specificity was 0.93 (95% CI  =  0.88–0.97). The area under the summary receiver operating characteristic (SROC) curve was 0.8731. However, on a per-lesion basis, the sensitivity and specificity of NBI were 0.69 (95% CI  =  0.63–0.74) and 0.91 (95% CI  =  0.87–0.94), respectively. The SROC was 0.9009. The pooled sensitivity and specificity of magnification endoscopy (NBI-ME) were 0.76 (95% CI  =  0.61–0.87) and 0.89 (95% CI  =  0.80–0.94), respectively, on per-patient analysis. On a per-lesion basis, the pooled sensitivity and specificity of NBI-ME were 0.84 (95% CI  =  0.76–0.89) and 0.93 (95% CI  =  0.89–0.96), respectively. Heterogeneity was observed with an I^2^ for diagnostic odds ratio (DOR) of 0.01% and 85.8%, respectively. There was no statistical significance for the evaluation of publication bias.

**Conclusions:**

Our meta-analysis shows that NBI is a useful tool for differential diagnosis of GIM with relatively low sensitivity and high specificity.

## Introduction

Gastric cancer (GC) remains a major cancer burden across the globe [1]. Although the trend in death rates for GC is decreasing, these tumors continue to have a poor prognosis and few efficacious therapeutic options, particularly in advanced stages of cancer. It is now established that the pathogenesis of gastric cancer is a multifactorial process in which both environmental and related factors play vital roles [2]. It is a multistep process that includes the sequential development of chronic gastritis followed by mucosal atrophy with hyperchlorhydria and intestinal metaplasia (IM), dysplasia, and finally adenocarcinoma [2]–[4]. IM is generally considered as the “field cancerization” in the gastric mucosa. However, the frequency of this type of lesion is so low that it is not a traditional endoscopic finding typical of IM. Fortunately, improved endoscopic techniques have made possible not only the discovery of early gastric cancers but also the recognition of mucosal changes that precede malignant degeneration [5].

Narrow band imaging (NBI) is an optical image enhancement technology that uses two short wavelength light beams that are 415 nm (blue) and 540 nm (green) [6]. It is an endoscopic imaging technique for the enhanced visualization of microvascular architecture and microsurface structure between the epithelial surface and subjacent vascular pattern [7]. Several studies have reported a correlation between the endoscopic mucosal pattern observed in the gastric mucosa with NBI endoscopy [8]–[10], investigating a diagnostic rate correlation between NBI appearances and pathology in GC. Previous studies using the NBI system with magnification endoscopy (NBI-ME) in the gastric mucosa showed that the appearance of a light blue crest in the mucosa is a distinctive endoscopic finding that suggests an increased likelihood of detecting GIM in the stomach [11]. More precisely, blue-white patchy areas are often observed in NBI images of the antrum in patients with gastric intestinal metaplasia (GIM).

In this work, we performed a meta-analysis of published data to assess the overall diagnostic accuracy (sensitivity and specificity) of NBI for differential diagnosis of GIM in the gastric.

## Materials and Methods

### Search strategy

We systematically searched the Medline, Cochrane Library databases and EMBASE for all articles on the association NBI and GC studies published until November 2013, by using the following search terms: “gastric” (or “stomach”), “narrow band imaging” and “NBI.” The reference lists of all the retrieved articles were examined to identify any additional articles missed during the initial search. Two investigators independently searched and extracted the data; disagreements were resolved by discussion. When necessary, we contacted the authors for detailed information. Only studies on humans and in English language were considered for inclusion.

### Inclusion and exclusion criteria

The inclusion criteria were as follows:

1. Studies that used NBI for gastric diseases;
2. Diagnostic clinical trials that evaluated the accuracy of NBI for differential diagnosis of GIM;
3. Studies that compared NBI with histology as the gold standard;
4. Studies with available data for constructing contingency tables for true positive (TP), false positive (FP), false negative (FN) and true negative (TN) determination; and
5. Studies that were published as a full article.

The exclusion criteria were as follows:

1. Data without histological confirmation of lesions;
2. Studies with incomplete data;
3. Studies that overlapped the studies selected (i.e., studies from the same study group, institution, and period of inclusion); and
4. Letters, editorials and expert opinions, review without original data, case reports or studies with fewer than 20 cases.

### Data extraction

Two reviewers independently extracted data by using a standardized form. If there was inconsistency, the original papers were retrieved and jointly investigated to resolve the disagreement. TP, FP, FN and TN were extracted using the histological findings as gold standard.

We constructed 2×2 tables that contained the number of GIM. The data were extracted either on a ‘per-patient’ or a ‘per-lesion’ element when possible. We also extracted the first author, publication year, region, patient's age, sex ratio, number of lesions, type of study, histological reference standard, reference test, number of endoscopist and endoscopes used.

### Qualitative assessment

To assess study quality and potential for bias, we used the Quality Assessment of Diagnostic Accuracy Studies-2 (QUADAS-2) tool [12]. The QUADAS-2 tool is completed on 4 key domains that are rated in terms of the risk of bias: patient selection, index test, reference standard, and flow and timing. If a study is judged as “low” on all domains relating to bias or applicability, then it is appropriate to have an overall judgment of “low risk of bias” or “low concern regarding applicability” for that study. If a study is judged “high” or “unclear” in 1 or more domains, then it may be judged “at risk of bias” or as having “concerns regarding applicability”. Quality assessment of the included studies was performed and crosschecked independently by two reviewers.

### Statistical analysis

We performed data analysis using Meta-DiSc (version 1.4) software [13]. The sensitivity and specificity of NBI in each study were extracted or calculated using 2×2 contingency tables of lesion diagnosis. Ninety-five percent confidence intervals for sensitivity, specificity, and predictive values were also calculated. The joint distribution of true-positive rate (TPR) and false-positive rate (FPR) was analyzed with a summary receiver operating characteristic (SROC) curve [14]–[16]. Values for the diagnostic odds ratio (DOR), Q-statistic, and area under the ROC curve (AUC) were used to analyze the diagnostic precision of NBI. A higher DOR suggests that the diagnostic precision of NBI is greater [17]. Most clinical tests have an AUC value between 0.5 and 1.0, with a better diagnostic precision correlating with an AUC closer to 1.0 [18].

Heterogeneity in meta-analysis refers to a high degree of variability in study results, a fairly common finding in diagnostic meta-analyses. In the presence of significant heterogeneity, pooled, summary estimates from meta-analyses are not meaningful. The Q-statistic is a form of the chi-squared test that measures heterogeneity between studies. All of the p values were two-sided.

Finally, potential publication bias was investigated using Begg's funnel plot and Egger's regression test [19], [20]. All analyses were performed using STATA software, version 11.0 (STATA, College Station, TX). All of the p values were two-sided.

## Results

### Eligible studies

**Figure 1:**
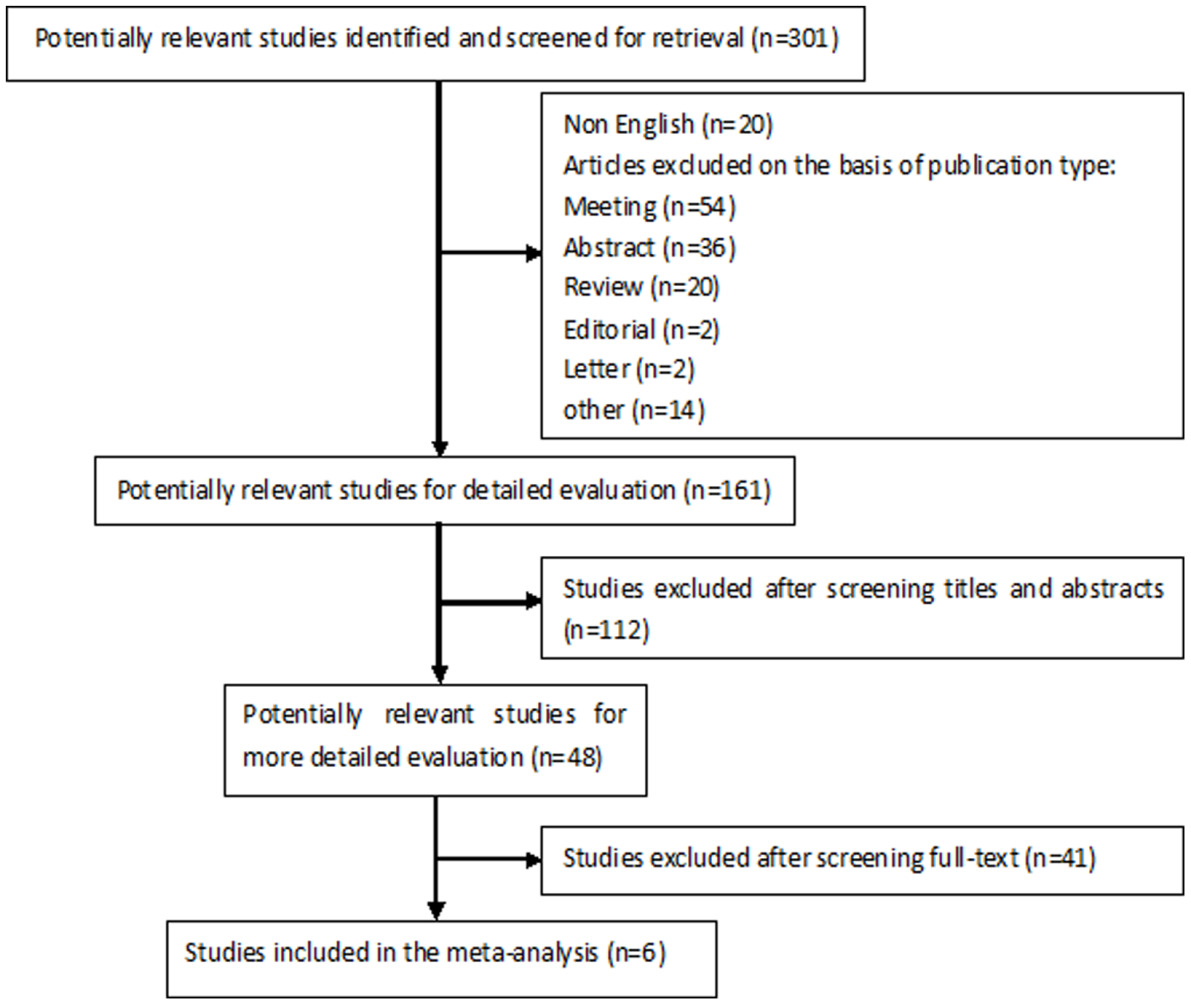
Flow chart showing the process for selecting eligible studies in this meta-analysis.

Figure 1 shows the six eligible studies identified from the literature. The main characteristics of the studies are reported in Table 1. Overall, 347 patients were enrolled, with a mean of 58 patients per study (range: 34–100 patients). Data for evaluating the accuracy of NBI for differential diagnosis of GIM were extracted from these studies.

**Table 1:** Characteristics of the included studies.

Author (year)	Location	Number of subjects, n	Lesions examined, n	Mean age, years	M/F	Study design	Histological reference standard	Reference test	Endoscopists number, n	NBI system	Magnifying
Edoarda et al [21] 2013	Italy	100	–	67	42/58	Prospective	GIM	random biopsy sample	3	GIF-Q160Z	Y
Pimentele-Nunes et al [22] 2012	Portugal	85	255	61	50/35	Prospective	GIM dysplasia	random biopsy sample	3	GIF-180	U
Rerknimitr et al [23] 2010	Thailand	38	228	59.5	20/18	Prospective	GIM	random biopsy sample	2	GIF-Q160Z	Y
Capelle et al [24] 2010	Netherlands	43	121	58.7	28/15	Prospective	GIM dysplasia	random biopsy sample	1	GIF-Q180	U
An et al [25] 2012	Korea	47	94	55	24/23	Prospective	GIM	random biopsy sample	1	GIF-H260Z	Y
Uedo et al [26] 2006	Japan	34	219	65	24/10	Prospective	GIM	random biopsy sample	1	GIF–Q240Z	Y

GIM, gastric intestinal metalasia; Y, yes; U, unmentioned.

Out of the six studies included, four studies gave details of GIM characterization on a per-patient basis [21]–[24] and four studies yielded data for per-lesion analysis [22], [24]–[26]. The details of the included studies are summarized in Table 1.

### Quality assessment

The quality of the eligible studies according to the QUADAS-2 criteria is shown in Table 2. Generally, the included studies met most of the quality criteria. Among the seven studies, only two enrolled patients were previously diagnosed by endoscopic biopsy [23], [24]. The endoscopic examinations in three studies were performed by only one experienced endoscopist [24], [25], [26]. These factors may all provide a risk of bias.

**Table 2:** The Quality Assessment of Diagnostic Accuracy Studies-2 (QUADAS-2) tool for quality assessment of studies selected for the meta-analysis.

Study	Risk of Bias				Applicability Concerns		
	Patient Selection	Index Test	Reference Standard	Flow and Timing	Patient Selection	Index Test	Reference Standard
Edoarda et al [21]	L	L	L	L	L	L	L
Pimentele-Nunes et al [22]	L	L	L	L	L	L	L
Rerknimitr et al [23]	H	L	L	L	H	L	L
Capelle et al [24]	H	H	L	L	H	L	L
An et al [25]	L	H	L	L	L	L	L
Uedo et al [26]	L	H	L	L	L	L	L

L, low risk; H, high risk; U, unclear risk.

### Diagnostic accuracy of NBI on a per-patient basis

**Figure 2:**
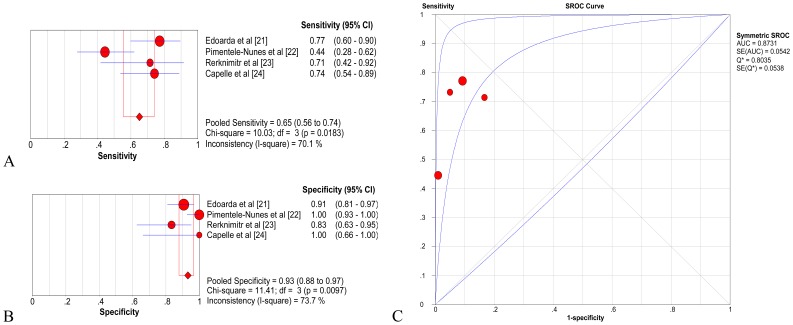
Gastric intestinal metaplasia (GIM), per-patient analysis of diagnostic performance of narrow band imaging (NBI). A. Pooled sensitivity for NBI to differentiate GIM; B. Pooled specificity for NBI to differentiate GIM; C. The summary receiver operating characteristic (SROC) cure for diagnosis by NBI. CI, confidence interval; df, degrees of freedom; AUC, area under curve; SE, standard error.

Four studies including a total of 266 patients were analyzed for NBI on a per-patient basis [21]–[24]. The pooled sensitivity and specificity of NBI were 0.65 (95% CI  =  0.56–0.74) and 0.93 (95% CI  =  0.88–0.97), respectively (Fig. 2A, 2B). The pooled positive likelihood ratio (LR) was 7.55 (95% CI  =  3.57–15.98), and the pooled negative LR was 0.36 (95% CI  =  0.22–0.59). The pooled DOR was 28.06 (95% CI  =  12.02–65.55) using a random-effects model. The AUC was 0.8731 (SE  =  0.0542) with Q*  =  0.8035 (SE  =  0.0538) (Fig. 2C), indicating a high level of diagnostic accuracy for NBI on a per-person basis.

**Figure 3:**
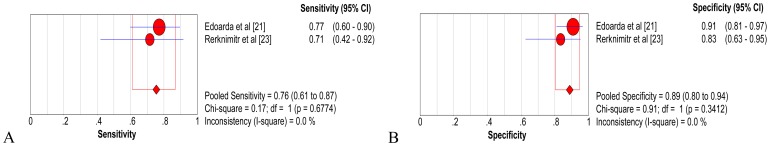
Gastric intestinal metaplasia (GIM), per-patient analysis of diagnostic performance of magnification narrow band imaging (NBI-ME). A. Pooled sensitivity for NBI-ME to differentiate GIM; B. Pooled specificity for NBI-ME to differentiate GIM; C. The summary receiver operating characteristic (SROC) cure for diagnosis by NBI-ME. CI, confidence interval; df, degrees of freedom; AUC, area under curve; SE, standard error.

Two studies [21], [23] including a total of 138 patients were analyzed for NBI-ME on a per-patient basis. The pooled sensitivity and specificity of NBI-ME were 0.76 (95% CI  =  0.61–0.87) and 0.89 (95% CI  =  0.80–0.94), respectively (Fig. 3A, 3B). The pooled positive likelihood ratio (LR) was 6.33 (95% CI  =  3.30–12.14), and the pooled negative LR was 0.28 (95% CI  =  0.17–0.46). The pooled DOR was 23.65 (95% CI  =  9.32–59.99) using a random-effects model.

### Diagnostic accuracy of NBI on a per-lesion basis

**Figure 4:**
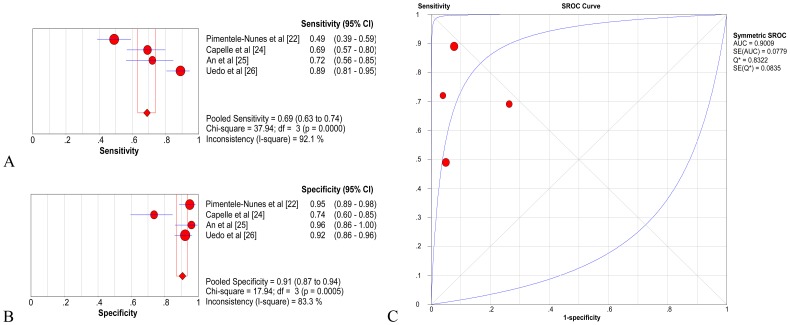
Gastric intestinal metaplasia (GIM), per-lesion analysis of diagnostic performance of narrow band imaging (NBI). A. Pooled sensitivity for NBI to differentiate GIM; B. Pooled specificity for NBI to differentiate GIM; C. The summary receiver operating characteristic (SROC) cure for diagnosis by NBI. CI, confidence interval; df, degrees of freedom; AUC, area under curve; SE, standard error.

In the pooled estimates for biopsy level analysis, based on evidence from four studies including 209 people and 689 lesions, the pooled sensitivity and specificity of NBI were 0.69 (95% CI  =  0.63–0.74) and 0.91 (95% CI  =  0.87–0.94), respectively (Fig. 4A, 4B) [22], [24]–[26]. The pooled positive LR was 7.88 (95% CI  =  2.93–21.15), and the pooled negative LR was 0.31 (95% CI  =  0.16–0.60). The pooled DOR was 27.31 (95% CI  =  7.10–105.04) using a random-effects model. The AUC was 0.9009 (SE  =  0.0779) with Q * = 0.8322 (SE  =  0.0835), indicating a high level of diagnostic accuracy for NBI (Fig. 4C).

**Figure 5:**
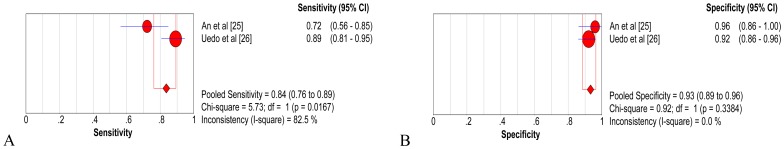
Gastric intestinal metaplasia (GIM), per-lesion analysis of diagnostic performance of magnification narrow band imaging (NBI-ME). A. Pooled sensitivity for NBI-ME to differentiate GIM; B. Pooled specificity for NBI-ME to differentiate GIM; C. The summary receiver operating characteristic (SROC) cure for diagnosis by NBI-ME. CI, confidence interval; df, degrees of freedom; AUC, area under curve; SE, standard error.

Based on evidence from two studies, including 81 people and 313 lesions, the pooled sensitivity and specificity of NBI-ME were 0.84 (95% CI  =  0.76–0.89) and 0.93 (95% CI  =  0.89–0.96), respectively (Fig. 5A, 5B) [25], [26]. The pooled positive LR was 12.26 (95% CI  =  7.08–21.24), and the pooled negative LR was 0.19 (95% CI  =  0.07–0.48). The pooled DOR was 85.50 (95% CI  =  38.66–189.06) using a random-effects model.

### Test for heterogeneity

On a per-patient basis, heterogeneity was observed with an I^2^ value of 0.01% for the DOR. Heterogeneity was observed among studies that were pooled for a per-lesion analysis, with an I^2^ value of 85.8% for the DOR. I^2^ values of 25%, 50% and 75% may be considered to represent low, moderate and high inconsistency [27]. There was low heterogeneity among studies that were pooled together for a per-patient analysis. Nevertheless, there was high heterogeneity for a per-lesion analysis.

We used metaregression and subgroup analysis to identify the source of heterogeneity. The following factors were analyzed for the metaregression analysis number of patients (< 50 or ≥ 50), number of lesions examined (< 200 or ≥ 200) and number of endoscopists (< 2 or ≥ 2). The meta-regression did not show any relationship between the characteristics of the studies and the diagnostic odds ratio (Table 3).

**Table 3:** Meta-regression for the potential source of heterogeneity.

Study characteristic	Coefficient	*P* value	Relative DOR (95% CI)*
Number of patient (<50 vs. ≥50)	3.689	0.3381	0.38 (0.00–31315089157159.60)
Number of lesion (<200 vs. ≥200)	3.771	0.2996	1.49 (0.00–297479.11)
Number of endoscopists (<2 vs. ≥2)	4.313	0.0029	0.31(0.04–2.14)

CI, confidence interval;*Relative diagnostic odds ratio (OR) is <1 when studies with the characteristic produce a lower diagnostic OR and >1 when the reverse is true.

### Publication bias estimate

**Figure 6:**
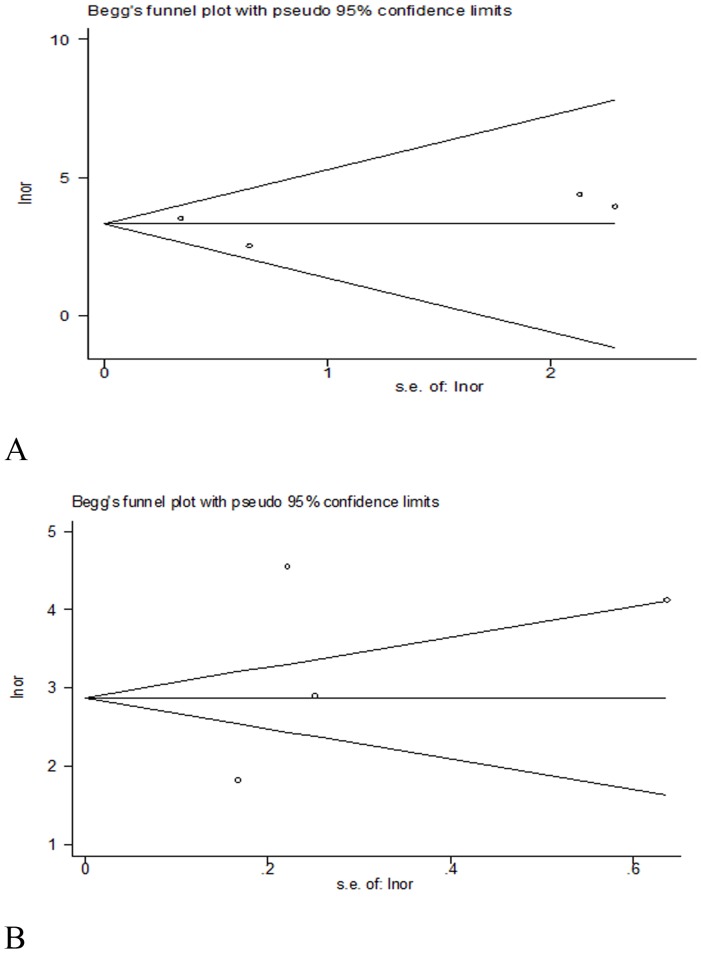
Funnel plot was constructed by Begg's and Egger's test to evaluate publication bias. A. Begg's test for the studies differentiating GIM on a per-patient analysis; B. Begg's test for the studies differentiating GIM on a per-lesion analysis. Lnor, ln [(TP*TN)/(FP*FN)]; s.e.of:lnor, (1/TP)+(1/FP)+(1/FN)+(1/TN).

We used Begg's funnel plot and Egger's test to address potential publication bias in the available literature. The Begg's test indicated a p value of 0.734 for the studies differentiating GIM on a per-patient analysis and a p value of 0.734 on a per-lesion analysis (Fig. 6A). The Egger's test gave a value of 0.06 (95% CI  =  −3.605948 to 3.714032, p =  0.955) on a per-patient analysis and 0.72 (95% CI  =  −30.81397 to 43.18541, p =  0.547) on a per-lesion analysis (Fig. 6B). These results suggest that there was no statistical significance in the evaluation of publication bias.

## Discussion

GC is one of the most prevalent and lethal malignancies worldwide due to the difficulty of early detection and high postsurgical recurrence rate [28]. Patients afflicted with GC are often asymptomatic, and there is a lack of sensitive and reliable biomarkers for early detection of GC. GIM may reveal sings of the development of intestinal-type gastric cancer [29]. The NBI technique is based on a modification of the spectral characteristics of the optical filter in the light source, leadings to improved visibility of mucosal structures [6]. Therefore, an NBI endoscopic technique has made possible the discovery of mucosal changes that precede malignant changes.

This meta-analysis is the first to summarize the available evidence regarding the diagnostic performance of NBI for the differential diagnosis of GIM. In addition, Begg's and Egger's bias indicators showed no significant publication bias in a per-patient element or a per-lesion element (p> 0.05).

In this meta-analysis, a high level of diagnostic precision was achieved for NBI-based characterization of GIM. The overall sensitivity of NBI for diagnosing GIM was 0.65 (95% CI  =  0.56–0.74) with an overall specificity of 0.93 (95% CI  =  0.88–0.97) on a per-patient basis. Moreover, NBI has a sensitivity and specificity of 0.69 (95% CI  =  0.63–0.74) and 0.91 (95% CI  =  0.87–0.94), respectively, on a per-lesion element. These data indicates that NBI has a high level of diagnostic accuracy for GIM.

The NBI system is a unique sequential electronic endoscopy system, one of the greatest advantages of this system is its ability to visualize the minute mucosal surface without the need for chromoendoscopy [30]. NBI-ME is useful for diagnosing the depth of invasion and abnormal vascular patterns than NBI without magnification [31]. Diagnostic precision of GIM may be different with or without magnification. Therefore, we analyzed the sensitivity and specificity of NBI with magnification. The pooled sensitivity and specificity of NBI-ME were 0.76 (95% CI  =  0.61–0.87) and 0.89 (95% CI  =  0.80–0.94), respectively, on per-patient analysis. On a per-lesion basis, the pooled sensitivity and specificity of NBI-ME were 0.84 (95% CI  =  0.76–0.89) and 0.93 (95% CI  =  0.89–0.96), respectively. These data indicated that NBI with magnification might have a much higher level of diagnostic accuracy for GIM than NBI without magnification.

Heterogeneity was observed among studies that were pooled on a per-patient analysis of GIM characterization, with an I^2^ value of 0.01% for the DOR. However, the I^2^ value was 85.8% on a per-lesion analysis. As a result, there was a high heterogeneity for per-lesion analysis. Such heterogeneity could be due to variation in thresholds, disease spectrum, test methods, and study quality among the selected studies. In this study, we performed a meta-regression analysis to estimate the effect of study characteristics, e.g., the number of enrolled patients (< 50 or ≥ 50), number of lesions examined (< 200 or ≥ 200) and number of endoscopists (< 2 or ≥ 2). However, as shown in Table 3, these factors have no influence on heterogeneity. The reason for the formation of heterogeneity may arise from the study quality. We need more high quality data to account for this possibility.

Our study had some limitations. First, the meta-analysis included only six studies. Further analysis with high quality data and data from multicenter studies is necessary to evaluate whether NBI yields adequate results in the detection of GIM. Second, the NBI endoscopic procedure was performed by expert endoscopists. However, three studieswere performed with one expert endoscopist [24]–[26]. Thus, the detection of GIM by NBI could possibly be biased by the endoscopist. Third, there was heterogeneity between studies on a per-lesion basis. The random-effect model was used to summarize the effects of NBI. Although we performed meta-regression and subgroup analysis to identify the sources of heterogeneity, we did not determine the source of the heterogeneity. Fourth, the TP, FP, TN and FN could not be extracted from the sensitivity and specificity calculation in three related studies [32]–[34]. Lastly, we included only studies published in English; therefore, language bias may exist. Some useful information may have been missed in this review.

In this study, we also searched the data to evaluate the accuracy of NBI for the diagnosis of intraepithelial neoplasia (IN). These studies showed that NBI detected more lesions, including low-grade IN and high-grade IN [35]–[38]. The sensitivity and specificity of NBI for diagnosing low-grade IN were 69.57% and 89.83%, respectively. Additionally, the sensitivity and specificity of NBI for high-grade IN were 89.63% and 69.57%, respectively [38]. The accuracy of NBI to diagnose IN was 81% (95% CI  =  69%–93%) [36]. These data indicated that NBI has a high level of diagnositic accuracy for IN. Unfortunately, there is not enough information extracted to evaluate diagnostic accuracy. IN is one part of the multistep process to develop GC. However, these results still suggests that NBI has a high diagnostic accuracy for GC. In brief, the existing evidence shows that NBI is an effective method for the identification of early gastric cancer.

## Conclusions

In conclusion, our meta-analysis showed that NBI was an accurate and useful tool to diagnose GIM with low sensitivity and high specificity, especially for magnifying NBI. However, as only a few studies were available, we believe that more general information with high quality trials should be provided to update this study.

## Supporting Information

Checklist S1 **PRISMA Checklist.**
(DOC)
